# Improvement of Oxidative and Metabolic Parameters by Cellfood Administration in Patients Affected by Neurodegenerative Diseases on Chelation Treatment

**DOI:** 10.1155/2014/281510

**Published:** 2014-07-10

**Authors:** Alessandro Fulgenzi, Rachele De Giuseppe, Fabrizia Bamonti, Maria Elena Ferrero

**Affiliations:** ^1^Department of Biomedical Sciences for Health, University of Milan, Via L. Mangiagalli 31, 20133 Milan, Italy; ^2^Department of Biomedical, Surgical and Dental Sciences, University of Milan and Haematology-Oncology and BMT Unit, IRCCS Ca' Granda Ospedale Maggiore Policlinico, Via F. Sforza 33, 20122 Milan, Italy

## Abstract

*Objective*. This prospective pilot study aimed at evaluating the effects of therapy with antioxidant compounds (Cellfood, and other antioxidants) on patients affected by neurodegenerative diseases (ND), who displayed toxic metal burden and were subjected to chelation treatment with the chelating agent calcium disodium ethylenediaminetetraacetic acid (CaNa_2_EDTA or EDTA). *Methods*. Two groups of subjects were studied: (a) 39 patients affected by ND and (b) 11 subjects unaffected by ND (controls). The following blood parameters were analyzed before and after three months' treatment with chelation + Cellfood or chelation + other antioxidants: oxidative status (reactive oxygen species, ROS; total antioxidant capacity, TAC; oxidized LDL, oxLDL; glutathione), homocysteine, vitamin B12, and folate. *Results*. After 3-months' chelation + Cellfood administration oxLDL decreased, ROS levels were significantly lower, and TAC and glutathione levels were significantly higher than after chelation + other antioxidants treatment, both in ND patients and in controls. Moreover, homocysteine metabolism had also improved in both groups. *Conclusions*. Chelation + Cellfood treatment was more efficient than chelation + other antioxidants improving oxidative status and homocysteine metabolism significantly in ND patients and controls. Although limited to a small number of cases, this study showed how helpful antioxidant treatment with Cellfood was in improving the subjects' metabolic conditions.

## 1. Introduction

The influence of nutrition and dietary supplements on the course of neurodegenerative diseases (ND) has been recently studied, particularly, the effects of nutritional factors, such as polyunsaturated fatty acids, vitamins, milk proteins, gluten, and probiotics, on the development, relapse rate, and progression of multiple sclerosis (MS) [1]. Recently malnutrition at the time of diagnosis has been associated with a shorter duration of disease in amyotrophic lateral sclerosis (ALS) [2]. Nutritional approaches have been proposed to reduce the risk and improve the management of Alzheimer's disease (AD) [3]. Cell damage due to both oxidative stress and depletion of endogenous antioxidants could be considered mechanisms of injury for ND. Indeed, the use of antioxidants seems to help prevent the formation of reactive species and counteract the damage to DNA, lipids, proteins, and other biomolecules [4]. For example, serum nitric oxide and peroxynitrite levels have been shown to be higher in patients affected by Parkinson's disease (PD) [5] than in controls, establishing a relationship between serum levels of these oxidants and the severity of the disease. In this context, other studies have demonstrated the usefulness of phenolic compounds and N-acetylcysteine as antioxidants in ND and PD, respectively [6, 7].

Mitochondrial dysfunction is considered to be the basis of the development and progression of several neurologic diseases with different aetiologies, including ND [8]. Therefore, mitochondria have become an interesting target of drug therapy [9]. Particularly, as reported by Mao et al.'s study, mitoQ, a mitochondria-targeted antioxidant, can delay disease progression and alleviate pathogenesis in an experimental autoimmune encephalomyelitis mouse model of MS [10].

We had previously shown how helpful antioxidant compound Cellfood was in improving mitochondrial respiratory metabolism of endothelial cells and inhibiting hypoxia-induced reactive oxygen species (ROS) generation* in vitro* [11]. In this prospective study we evaluated the influence of Cellfood treatment on blood parameters in patients affected by ND and subjected to chelation therapy with the chelating agent calcium disodium ethylenediaminetetraacetic acid (CaNa_2_EDTA or EDTA), administered intravenously. Two groups of subjects were studied: 39 patients affected and 11 subjects unaffected by ND (controls). All subjects were affected by chronic body burden of heavy metals and were treated with EDTA to remove metal intoxication [12–14]. The subjects were also daily treated with antioxidant therapy to help the detoxification process: some of them received Cellfood and some other antioxidants [15].

Oxidative status represents the result of the balance between ROS generation and antioxidant capacity of the organism. Blood parameters could possibly highlight oxidative stress conditions. In this study serum concentrations of ROS, total antioxidant capacity (TAC), oxidized LDL (oxLDL), cholesterol profile (total cholesterol (TC); HDL; and LDL), and the blood GSH/GSSG red-ox couple were assessed. The latter is mostly responsible for maintaining homeostasis of cell red-ox state [16]. Homocysteinemia and metabolically related vitamins (vitamin B12, active vitamin B12, serum, and erythrocyte folate) were also determined. In fact, if vitamin levels are inadequate, hyperhomocysteinemia can have a prooxidant effect causing or promoting oxidation.

The aim of this prospective pilot study was to evaluate the influence of Cellfood treatment during chelation therapy and to compare it with that of other antioxidants.

## 2. Materials and Methods

### 2.1. Patient Recruitment

Out of 80 consecutive subjects who had undergone a medical checkup in an outpatient medical center, only 50 were selected and enrolled for this study due to their compliance in following the protocol, for example, receiving chelation therapy once a week by personal choice and taking daily antioxidants. Antioxidants were distributed at random.

Twenty patients were affected by MS; fifteen of them had been previously treated with conventional drugs against MS (e.g., immunosuppressant agents, such as mitoxantrone and azathioprine, broad-spectrum immunomodulatory agents, such as glatiramer acetate and interferon *β*, monoclonal antibodies, such as rituximab and natalizumab, and the recently discovered fingolimod, a sphingosine-1-phosphate-receptor modulator) [17, 18]. However, all these patients had interrupted previous therapies almost 2 months before starting chelation treatment. Five of these MS patients had never been previously treated with drugs.

Nineteen patients affected by ND were also recruited as well as 11 subjects not affected by any known disease but previously exposed to environmental or occupational heavy metals, who decided to start chelation therapy and acted as controls. Subjects' age ranged from 18 to 75.

All subjects provided written informed consent to participate in this study. Declaration of Helsinki and all procedures involving human participants were approved by the Milan University's Ethical Advisory Committee (number 64/14).

### 2.2. Study Design

All subjects (ND and controls) underwent chelation therapy for 3 months. EDTA is endowed with antioxidant properties; in fact, without any added vitamin C, it can decrease oxidative DNA damage and lipid peroxidation [19]. However, since its administration occurred once a week, the subjects were treated daily with antioxidants. At the beginning (basal values) and at the end of the treatments (after 3 months), blood lipid panel, homocysteine metabolism, and some oxidative stress parameters were evaluated.

### 2.3. Chelation Test

All ND patients and controls had been subjected to “chelation test” in order to verify their possible burden by toxic metals. Generally, for the “chelation test,” EDTA (2 g), diluted in 500 mL physiological saline (Farmax srl, Brescia, Italy), is slowly (in about 2 hours) administered intravenously in subjects who are invited to collect urine samples before and after the first intravenous EDTA treatment. Urine collection following chelation treatment lasted 12 hours. Urine samples are accurately enveloped in sterile vials and sent to the Laboratory of Toxicology (Doctor's Data Inc., St. Charles, IL, USA) to be analysed, as previously reported [12]. Briefly, samples are acid-digested with certified metal-free acids (digestion takes place in a closed-vessel microwave digestion system), diluted with ultrapure water, and carried out via inductively coupled plasma with mass spectrometry (ICP-MS) utilizing collision/reaction cell methods coupled with ion-molecule chemistry, a reliable new method for reducing interference. Urine standards, both certified and in-house, are used for quality control and validation of data. To avoid the potentially great margin of error due to fluid intake and sample volume, results were reported in micrograms (*μ*g) per g of creatinine.

When the first “chelation test” showed intoxication by heavy metals, our subjects started chelation therapy once a week.

### 2.4. Antioxidant Supplementation

#### 2.4.1. Cellfood Treatment

Cellfood (Eurodream, La Spezia, Italy) is an antioxidant nutritional supplement containing 78 ionic/colloidal trace elements and minerals combined with 34 enzymes and 17 amino acids, all suspended in a solution of deuterium sulphate, efficient in protecting against oxidative damage* in vitro* [20]. A gradually increasing concentration of Cellfood was administered daily to subjects (22 ND patients and 6 controls) according to the following scheme: the first, second, and third day 1 drop in mineral water three times a day, the fourth, fifth, and sixth day 2 drops, the seventh and eighth day 3 drops three times a day, that is, 1 drop more three times a day, and finally 20 drops altogether were given three times a day. The treatment lasted three months.

#### 2.4.2. Other Antioxidant Treatments

Twenty-two patients (17 ND and 5 controls) took daily other antioxidants, instead of Cellfood. Particularly, 10 of them (6 ND and 4 controls) took *α*-lipoic acid (400 mg/day), the other 10 glutathione (Ultrathione, 500 mg/day), alone or together with multivitamin complexes, aminoacid and mineral mixtures, or probiotics. Also these treatments lasted three months.

### 2.5. Evaluation of Blood Parameters 

#### 2.5.1. Sample Collection

Biochemical parameters were measured in blood drawn from patients before starting therapy with EDTA and antioxidants (basal values) and after three months.

Peripheral blood samples were collected after overnight fasting into preevacuated and light-protected tubes, with no additive or with EDTA, in order to evaluate oxidative status (ROS; TAC; oxLDL) and glutathione and homocysteine metabolism (homocysteine, Hcy; holotranscobalamin, active B12; serum folate, s-Fol; erythrocyte folate, ery-Fol).

Serum aliquots were used to measure ROS, TAC, oxLDL, active B12, and s-Fol concentrations while EDTA whole blood was used for glutathione and ery-Fol levels determination. The remaining EDTA whole blood sample was centrifuged within 30 minutes to obtain plasma for total Hcy determination.

All the aliquots, except for the one used for blood counting, were immediately frozen and stored at −80°C ready for assay.

A 12-hour urine sample was used for the “chelation test,” as previously described.

#### 2.5.2. Oxidative Status

Serum ROS expressed as Carratelli Units (UCarr), oxLDL concentrations, and TAC were measured by using a commercial enzyme-linked immunoabsorbent assay (ELISA, Mercodia, Uppsala, Sweden) on the EASIA reader (Medgenix Diagnostics, Fleurus, Belgium) and by using spectrophotometric commercial kits (dROMs test, Diacron International, Grosseto, Italy; OXY-adsorbent test, Diacron International, Grosseto, Italy) on F.R.E.E. analyzer (Free Radical Elective Evaluator analyzer, Diacron International, Grosseto Italy) (Diacron), respectively.

Total and free glutathione concentrations were assessed by HPLC method followed by fluorescent detection using a commercially available kit (Chromsystems Instruments & Chemicals, Munich, Germany). Total glutathione is the sum of oxidized (GSSG) and free (GSH) glutathione existing in the sample prior to reduction. Briefly, since chromatography can only determine GSH, GSSG present in the sample was converted into GSH by using a reduction reagent which reduced one GSSG molecule to 2 GSH molecules obtaining total glutathione. GSSG concentration was calculated by subtracting the GSH amount from the total glutathione. GSH/GSSG ratio was also calculated and used as an oxidative stress marker.

#### 2.5.3. Homocysteine Metabolism

Plasma Hcy levels were measured using homocysteine liquid enzymatic assay (Sentinel Diagnostics, Milan, Italy) on Modular P analyser (Roche Diagnostics, Indianapolis, IN, USA). Serum active B12, s-Fol, and ery-Fol concentrations were determined using the relevant Abbott Microparticle Enzyme Immunoassay (MEIA) kits (Holotranscobalamin-Active-B12 and Architect Folate, Abbott Laboratories, Abbott Park, IL, USA) on Architect analyser (Abbott).

#### 2.5.4. Lipid Panel

Serum total cholesterol (TC), high-density lipoprotein (HDL) cholesterol, and low-density lipoprotein (LDL) cholesterol concentrations were determined using the routine tests on Modular P analyser. Total/HDL cholesterol and LDL/HDL cholesterol ratios were calculated together with oxLDL/HDL and oxLDL/LDL ratios.

### 2.6. Statistical Analysis

Data were analyzed by analysis of variance (ANOVA) with the solution type as main factor. Post hoc comparisons were made using Tukey's honestly significant difference test (HSD).

## 3. Results

### 3.1. Patients' Characteristics

**Figure 1:**
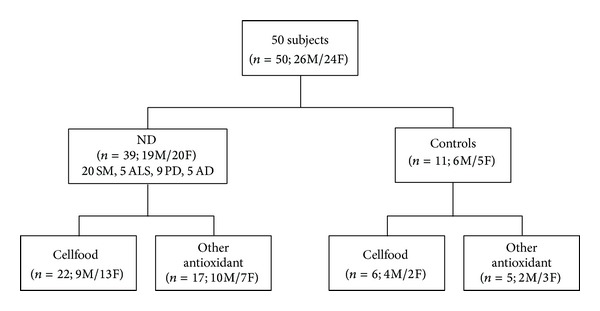
Scheme of enrolled subject's characteristics.

As shown in Figure 1, eleven of these subjects were classified as controls (C) because they were not affected by ND or other known diseases. Six of them took Cellfood and five took other antioxidants. Thirty-nine patients were classified as ND: 20 MS, 5 ALS, 9 PD, and 5 AD. Twenty-two of them took Cellfood while the other seventeen received other antioxidants. The subjects of these two groups were matched for age, sex, disease duration, and previous drug treatments. Mean age of each group was 43 ± 5. Subjects' basal values were obtained before the beginning of each treatment.

### 3.2. Chelation Therapy

The first “chelation test” of our subjects showed intoxication by heavy metals with prevalence of lead, cadmium, aluminium, and gadolinium (used as contrast agent in magnetic resonance imaging to diagnose MS) (data not shown). Consequently, the subjects were administered chelation therapy once a week.

### 3.3. Oxidative Status Parameters

**Figure 2:**
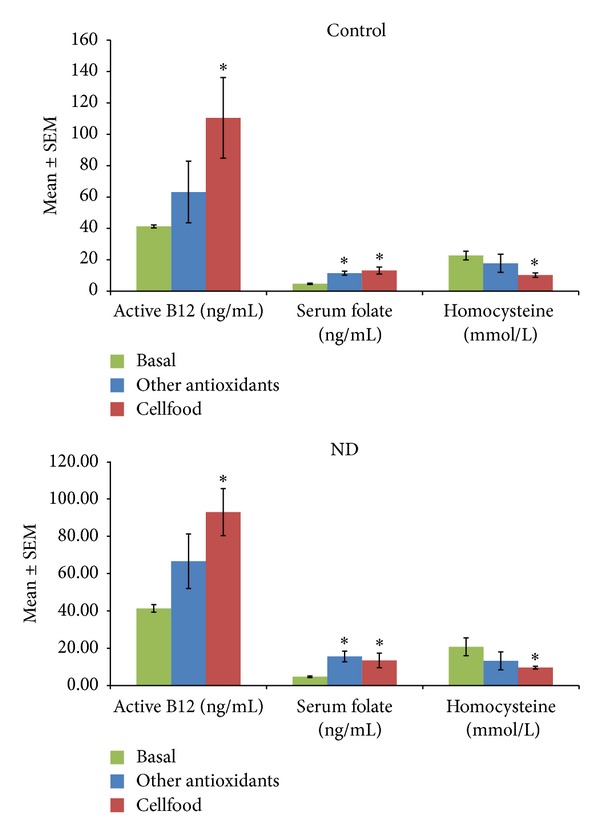
Serum active Vit B12, folate, and homocysteine levels measured in subjects unaffected (controls) or patients affected by neurodegenerative diseases (ND). All subjects' concentrations were determined before the beginning (basal values) and after three months of treatment with Cellfood or other antioxidants (see text). *P < 0.05 versus all basal values.

**Figure 3:**
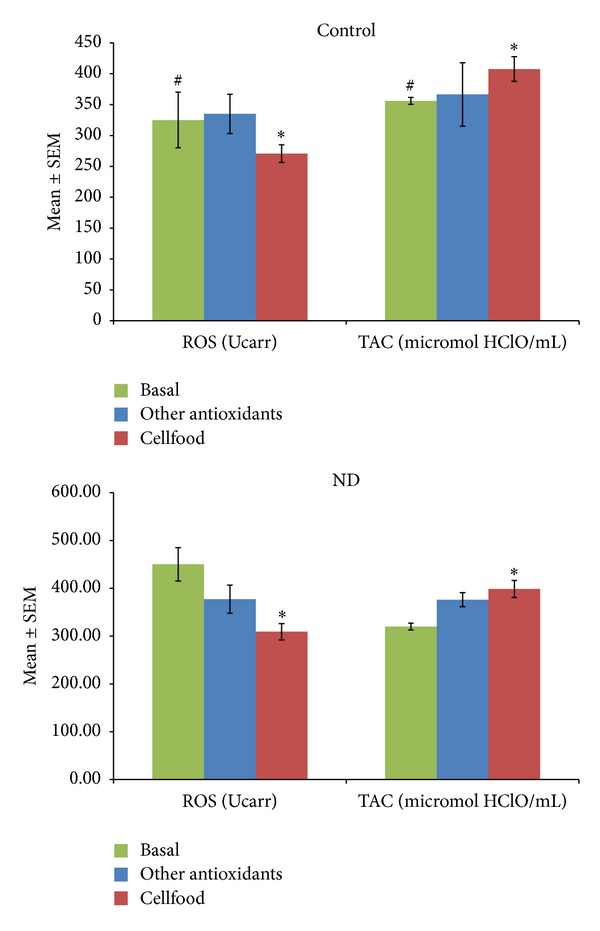
Serum reactive oxygen species (ROS) levels and total antioxidant capacity (TAC) measured in subjects unaffected (control) or patients affected by neurodegenerative diseases (ND). All subjects' concentrations were determined before the beginning (basal values) and after three months of treatment with Cellfood or other antioxidants (see text). *P < 0.05 versus all basal values; ^#^ *P* < 0.05 C versus ND (basal values).

**Figure 4:**
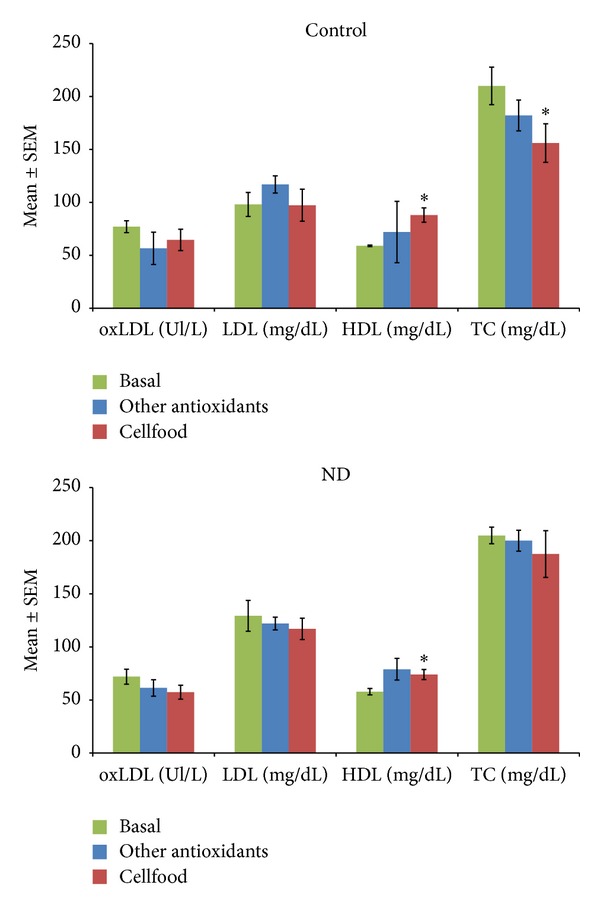
Serum oxidized LDL (oxLDL), LDL, HDL, and total cholesterol (TC) levels measured in subjects unaffected (controls) or patients affected by neurodegenerative diseases (ND). All subjects' concentrations were determined before the beginning (basal values) and after three months of treatment with Cellfood or other antioxidants (see text). *P < 0.05 versus all basal values.

**Figure 5:**
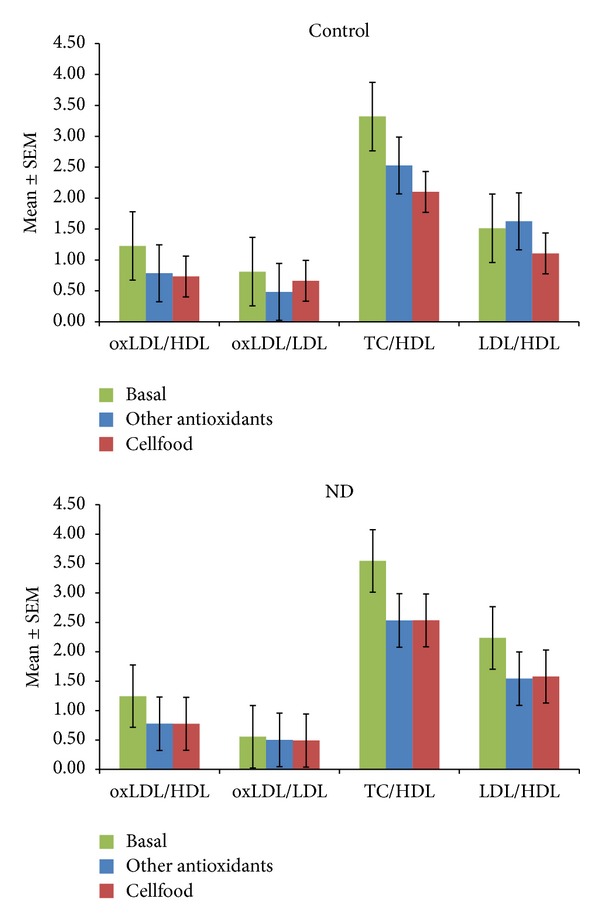
Oxidized LDL (oxLDL)/LDL ratios, oxLDL/LDL ratios, total cholesterol (TC)/HDL ratios, and LDL/HDL ratios calculated from the levels of subjects unaffected (controls) or patients affected by neurodegenerative diseases (ND). All subjects' values were determined before the beginning (basal values) and after three months of treatment with Cellfood or other antioxidants (see text).

Figure 2 shows the active B12, s-Fol, and Hcy concentrations as basal values and after 3 months' treatment (chelation + Cellfood or chelation + other antioxidants). After chelation + Cellfood treatment, low basal levels of active B12 improved significantly, both in controls and in ND patients; moreover, a significant decrease in Hcy levels but no significant variations in s-Fol concentrations were observed. Basal ROS levels were significantly higher and basal TAC levels were significantly lower in ND patients than in controls, as shown in Figure 3. Of note, in both controls and ND patients, only chelation + Cellfood treatment improved TAC and ROS values significantly compared with chelation + other antioxidants.  Figure 4 shows that cholesterol profile improved more in controls than in ND patients after both antioxidant treatments, while chelation + Cellfood treatment was significantly effective on both groups. Notably, also oxLDL levels decreased, even if not significantly so, in both groups. In addition, as reported in Figure 5, all ratios improved after both treatments in ND patients and in controls [21, 22].

**Figure 6:**
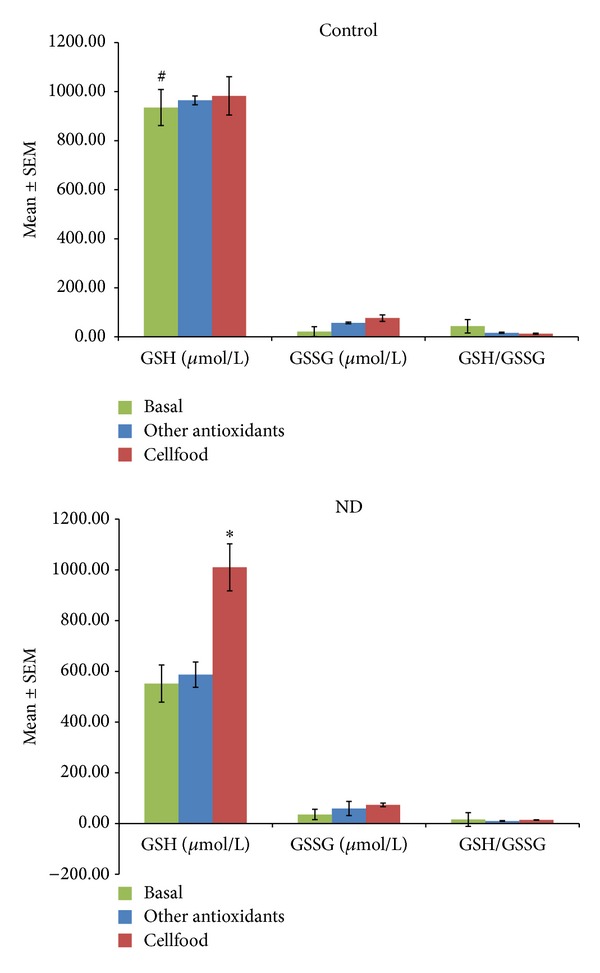
Free glutathione (GSH) and oxidized glutathione (GSSH) levels and GSH/GSSH ratios measured in blood obtained from subjects unaffected (control) or patients affected by neurodegenerative diseases (ND). All subjects' values were determined before the beginning (basal values) and after three months of treatment with Cellfood or other antioxidants (see text). *P < 0.05 versus all basal values; ^#^ *P* < 0.05 C versus ND (basal values).

High ROS levels were associated with low GSH values. At baseline, GSH levels were significantly higher in controls than in ND patients. Chelation + Cellfood treatment significantly increased GSH levels in ND patients (Figure 6). On the whole, our findings showed that chelation + Cellfood treatment was much more efficient than other antioxidant treatments.

## 4. Discussion

Much attention has been recently devoted to red-ox processes involving ROS in neurodegenerative diseases (ND) [23]. In particular, the protective role of antioxidants could possibly have clinical implications in PD, AD, ALS, and MS. Antioxidants contained in natural foods are really considered part of nutraceuticals, that is, compounds with a significant role in modifying and preserving healthy physiological functions [24]. Even if different ND may have unrelated pathophysiology, the role of ROS is recognised in the etiology of all ND. The effectiveness of nutritional antioxidants in some human diseases has been reported in this context [25, 26]. Therapies administrated to patients affected by serious ND cannot be limited to antioxidant integrators. The literature indicates that specific antioxidants can have chelating capacities and that a synergism exists between antioxidants and chelating agents [27].

To the best of our knowledge, ours is the first study showing the improvement in antioxidant capacity due to a treatment with antioxidants in subjects affected by chronic body burden of heavy metals onchelation therapy: some of them were affected by ND while others were unaffected by known diseases. We examined how oxidative and metabolic parameters can improve in ND patients. EDTA chelation therapy was administered to all the subjects recruited as required by urine chelation test results (data not shown). We measured blood levels of some oxidative and metabolic parameters before starting antioxidant treatments and three months after. Our results highlight how helpful chelation + antioxidant treatment was; *α*-lipoic acid or glutathione, whether or not associated with vitamins, aminoacids, minerals, and probiotics, was taken daily by about 50% of the subjects and Cellfood was taken by the remaining 50%.

Cellfood is a nutraceutical, antioxidant supplement containing natural trace elements and minerals combined with enzymes and amino acids, suspended in a solution of deuterium sulphate, efficient in protecting against oxidative damage [20]. In a previous study we showed that Cellfood treatment* in vitro *increased mitochondrial metabolism in endothelial cells [11]. In this study we showed that chelation + Cellfood was significantly more efficient than chelation + other antioxidants in modifying ND patients and controls oxidative and metabolic parameters. In fact, oxidative status parameters showed that chelation + Cellfood improved TAC and GSH values significantly by reducing ROS levels significantly and lowering oxLDL. Moreover, chelation + Cellfood improved cholesterol profile increasing HDL levels significantly. The effects of Cellfood were more evident in ND patients than in controls. In addition, chelation + Cellfood treatment increased active B12 and serum folate and reduced homocysteine levels significantly, as expected.

Some authors suggested that dietary polyphenols can protect subjects against ND. Indeed, olive polyphenol administration increased the levels of the nerve growth factor and brain-derived neurotrophic factor in mice brains [28]. Similarly, *α*-lipoic acid prevented damage induced by 6-hydroxydopamine or by chronic use of L-DOPA in dopaminergic neurons in a PD animal model [29]. A new glutathione derivative, endowed with an 8-hydroxyquinoline group as a metal-chelating moiety, displayed neuroprotective activities* in vitro* [30]. Some traditional vitamins seem to act as antioxidants on ND [31]. Moreover, a multivitamin supplement enriched with phytosterol reduced serum cholesterol in hyperlipidemic rats [32]. In addition, treatment with a novel oral nutraceutical formula containing omega-3 and omega-6 fatty acids and vitamins (vitamin A and vitamin E) reduced the risk of sustained disability progression without any serious adverse events in patients with relapsing remitting MS [33]. As mitochondrial dysfunction has been associated with the aging process and a large variety of human disorders such as ND [9], mitochondria became an interesting target for therapy. For instance, mitochondria-targeted antioxidants have been recently developed to treat PD [34].

Previous results indicated a correlation between elevated plasma cholesterol levels and cognitive impairment in AD patients [35]. In MS patients serum oxidized LDL concentrations have been proposed as a marker of clinical staging, since an increase in their levels was associated with expanded disability status scale [36]. Moreover, according to Kardys et al., high cholesterol affected retinal nerve fibre layer thickness in MS patients with optic neuritis [37]. In addition, as reported by Weinstock-Guttman et al., higher LDL levels associated with an increasing number of contrast agents caused ever increasing brain lesions in MS interferon beta-treated patients [38]. Therefore, the lowering of TC and TC/HDL ratios in our subjects treated with chelation + antioxidants suggests that these molecules could be given to MS patients. At baseline all our subjects' parameters of lipid profile generally were within normal values, and, interestingly, after a 3-month chelation + antioxidant treatment, there was a significant increase in HDL levels and decrease in TC concentrations in both groups on Cellfood. Moreover, according to Pawlak et al.'s report [21], lipoprotein ratios confirmed the beneficial effects of antioxidants in preventing the risk of cardiovascular diseases; this appeared more evident in controls than in ND patients. These ratios can help predict the degree of clinical benefits lowering risk levels well below the target of secondary prevention. These promising results are confirmed by the decreasing levels of oxLDL (an important biomarker of lipoprotein abnormalities and oxidative stress associated with atherosclerosis) and the decreasing oxLDL/LDL ratio (a new and more potent biomarker than standard lipid assessment) [21].

According to other authors' findings, homocysteine induces oxidative stress by promoting ROS production, by increasing NADPH oxidase, and by decreasing thioredoxin [39, 40]. Consequently, we decided to evaluate also Hcy metabolism.

Homocysteine, a metabolic intermediate of methionine, is the crucial aminothiol for the biosynthesis of other aminothiols (methionine and cysteine) and for cell red-ox balance (glutathione biosynthesis). Hyperhomocysteinemia has been implicated in the pathogenesis of atherosclerosis and is considered an independent marker of ischaemic stroke. A lack of vitamin B12 and/or folate leads to an accumulation of Hcy in the blood and is responsible for macrocytic anaemia and, often, irreversible neurological damage [41]. Neurological damage due to vitamin deficiency is quite a common condition: up to 75% of B12-deficient patients can have neurological or neuropsychiatric symptoms even if not anaemic. The earliest marker of a negative B12 balance is probably a low active B12 level (the complex vitamin B12-transcobalamin II) which is the critical transporter of cobalamin to peripheral tissues and can reflect vitamin B12 availability in the body [42]. In our study, after chelation + Cellfood treatment all subjects showed a significant increase in active B12 levels and a significant decrease in Hcy concentrations. Additionally, both chelation + antioxidant treatments increased folate concentrations significantly and helped control prooxidant molecule production. Chronic oxidative stress condition, in fact, could cause irreversible damage to cellular homeostasis.

Glutathione (GSH) is produced intracellularly from three amino acids (glutamate, cysteine, and glycine). GSH is oxidized to GSH disulfide (GSSG) by gluthatione peroxidase (GP) and then regenerated as GSH by the reaction with GSH reductase (GR) [43]. GSH plays a key role in cell resistance against oxidative damage by providing enzymes involved in ROS metabolism with reducing equivalents, by eliminating potentially toxic oxidation products, and by reducing oxidized protein thiols [44]. GSH availability under oxidative conditions is ensured by GSH recycling and biosynthetic pathways, which can be upregulated when oxidative stress occurs. Measurement of GSH and its disulfide forms (i.e., GSSG) in blood and their ratios is considered an index of the whole-organism oxidative status and a useful indicator of disease risk in humans [45]. Recently, Aoyama and Nakaki demonstrated that GSH function disorder is implicated in the aetiology of some neurodegenerative diseases such as Alzheimer's disease, Parkinson's disease, amyotrophic lateral sclerosis, progressive supranuclear palsy (PSP), Huntington's disease (HD), and multiple sclerosis [44]. According to this study, our ND patients showed lower GSH levels at baseline than controls. However, three-month treatment with chelation + Cellfood increased significantly GSH levels in ND patients.

Our subjects showed an oxidative stress condition probably due to accumulation of toxic metals in tissues which weaken the antioxidant system even in apparently healthy subjects (controls). Several studies indicate that exposure to heavy metals could affect the antioxidant potential of blood in people exposed to toxic elements. The mechanisms of metal-induced damage in mammalian systems include the production of ROS by altering cellular oxidative status of membranes and tissues and/or by directly lowering antioxidant reserves [46, 47]. Latency period (sometimes years) between a probable exposure to toxic substances and the onset of clinically evident ND depends on individual reactions and is not to be underestimated.

As concerns the exposure to toxic substances, during a prevention program in some Italian towns, we showed a decrease in TAC only in one population of a southern Italian village due to the high levels of arsenic in local spring water; accumulation of this toxic metal in tissues appeared to weaken the antioxidant defence system in apparently healthy subjects [48].

In conclusion, our study, though limited to a small number of cases, showed that easily detectable blood parameters can be a useful tool during chelation therapy with EDTA and antioxidant treatment.

These treatments can help counteract nutritional/environmental/occupational/pharmacological toxicity, if any.

Finally, chelation + Cellfood treatment was significantly more efficient than chelation + other antioxidant treatments in improving most parameters both in controls and in ND patients. This pilot study suggests that antioxidant treatment with Cellfood helps improve metabolic conditions.

## References

[1] von Geldern G, Mowry EM (2012). The influence of nutritional factors on the prognosis of multiple sclerosis. *Nature Reviews Neurology*.

[2] Limousin N, Blasco H, Corcia P (2010). Malnutrition at the time of diagnosis is associated with a shorter disease duration in ALS. *Journal of the Neurological Sciences*.

[3] Mi W, van Wijk N, Cansev M, Sijben JWC, Kamphuis PJGH (2013). Nutritional approaches in the risk reduction and management of Alzheimer's disease. *Nutrition*.

[4] Guerra-Araiza C, Álvarez-Mejía AL, Sánchez-Torres S (2013). Effect of natural exogenous antioxidants on aging and on neurodegenerative diseases. *Free Radical Research*.

[5] Kouti L, Noroozian M, Akhondzadeh S (2013). Nitric oxide and peroxynitrite serum levels in Parkinson's disease: correlastion of oxidative stress and the severity of the disease. *European Review for Medical and Pharmacological Sciences*.

[6] Ferreres F, Grosso C, Gil-Izquierdo A, Valentão P, Andrade PB (2013). Phenolic compounds from Jacaranda caroba (Vell.) A. DC.: approaches to neurodegenerative disorders. *Food and Chemical Toxicology*.

[7] Holmay MJ, Terpstra M, Coles LD (2013). N-acetylcysteine boosts brain and blood glutathione in gaucher and Parkinson diseases. *Clinical Neuropharmacology*.

[8] Kasote DM, Hegde MV, Katyare SS (2013). Mitochondrial dysfunction in psychiatric and neurological diseases: cause(s), consequence(s), and implications of antioxidant therapy. *BioFactors*.

[9] Edeas M, Weissig V (2013). Targeting mitochondria: strategies, innovations and challenges: the future of medicine will come through mitochondria. *Mitochondrion*.

[10] Mao P, Manczak M, Shirendeb UP, Reddy PH (2013). MitoQ, a mitochondria-targeted antioxidant, delays disease progression and alleviates pathogenesis in an experimental autoimmune encephalomyelitis mouse model of multiple sclerosis. *Biochimica et Biophysica Acta*.

[11] Ferrero E, Fulgenzi A, Belloni D, Foglieni C, Ferrero ME (2011). Cellfood improves respiratory metabolism of endothelial cells and inhibits hypoxia-induced rective oxygen species (ROS) generation. *Journal of Physiology and Pharmacology*.

[12] Fulgenzi A, Zanella SG, Mariani MM, Vietti D, Ferrero ME (2012). A case of multiple sclerosis improvement following removal of heavy metal intoxication. *BioMetals*.

[13] Lin-Tan DT, Lin JL, Yen TH, Chen KH, Huang YL (2007). Long-term outcome of repeated lead chelation therapy in progressive non-diabetic chronic kidney diseases. *Nephrology Dialysis Transplantation*.

[14] Lin J, Lin-Tan D, Hsu K, Yu C (2003). Environmental lead exposure and progression of chronic renal diseases in patients without diabetes. *The New England Journal of Medicine*.

[15] Uttara B, Singh AV, Zamboni P, Mahajan RT (2009). Oxidative stress and neurodegenerative diseases: a review of upstream and downstream antioxidant therapeutic options. *Current Neuropharmacology*.

[16] Presnell CE, Bhatti G, Numan LS (2013). Computational insights into the role of glutathione in oxidative stress. *Current Neurovascular Research*.

[17] O'Sullivan C, Dev KK (2013). The structure and function of the S1P1 receptor. *Trends in Pharmacological Sciences*.

[18] Chiba K, Adachi K (2012). Discovery of fingolimod, the sphingosine 1-phosphate receptor modulator and its application for the therapy of multiple sclerosis. *Future Medicinal Chemistry*.

[19] Roussel AM, Hininger-Favier I, Waters RS, Osman M, Fernholz K, Anderson RA (2009). EDTA chelation therapy, without added vitamin C, decreases oxidative DNA damage and lipid peroxidation. *Alternative Medicine Review*.

[20] Benedetti S, Catalani S, Palma F, Canestrari F (2011). The antioxidant protection of CELLFOOD against oxidative damage in vitro. *Food and Chemical Toxicology*.

[21] Pawlak K, Mysliwiec M, Pawlak D (2013). Oxidized low-density lipoprotein (oxLDL) plasma levels and oxLDL to LDL ratio—are they real oxidative stress markers in dialyzed patients?. *Life Sciences*.

[22] Millan J, Pinto X, Munoz A, Zuniga M, Rubies-Prat J, Pallardo LF (2009). Lipoprotein ratios: physiological significance and clinical usefulness in cardiovascular prevention. *Vascular Health and Risk Management*.

[23] Kovacic P, Somanathan R (2012). Redox processes in neurodegenerative disease involving reactive oxygen species. *Current Neuropharmacology*.

[24] Das L, Bhaumik E, Raychaudhuri U, Chakraborty R (2012). Role of nutraceuticals in human health. *Journal of Food Science and Technology*.

[25] Soory M (2012). Nutritional antioxidants and their applications in cardiometabolic diseases. *Infectious Disorders—Drug Targets*.

[26] Alvarez-Suarez JM, Giampieri F, Battino M (2013). Honey as a source of dietary antioxidants: structures, bioavailability and evidence of protective effects against human chronic diseases. *Current Medicinal Chemistry*.

[27] Patrick L (2003). Toxic metals and antioxidants: part II. The role of antioxidants in arsenic and cadmium toxicity. *Alternative Medicine Review*.

[28] De Nicoló S, Tarani L, Ceccanti M (2013). Effects of olive polyphenols administration on nerve growth factor and brain-derived neurotrophic factor in the mouse brain. *Nutrition*.

[29] de Araújo DP, De Sousa CN, Araújo PVP (2013). Behavioral and neurochemical effects of alpha-lipoic Acid in the model of Parkinson's disease induced by unilateral stereotaxic injection of 6-ohda in rat. *Evidence-Based Complementary and Alternative Medicine*.

[30] Cacciatore I, Cornacchia C, Fornasari E (2013). A glutathione derivative with chelating and in vitro neuroprotective activities: synthesis, physicochemical properties, and biological evaluation. *ChemMedChem*.

[31] Goldschmidt R, Arce PM, Khdour OM (2013). Effects of cytoprotective antioxidants on lymphocytes from representative mitochondrial neurodegenerative diseases. *Bioorganic and Medicinal Chemistry*.

[32] Csont T, Sarkozy M, Szucs G, Szucs C, Barkanyi J, Bencsik P (2013). Effect of a multivitamin preparation supplemented with phytosterol on serum lipids and infarct size in rats fed with normal and high cholesterol diet. *Lipids in Health and Disease*.

[33] Pantzaris MC, Loukaides GN, Ntzani EE, Patrikios IS (2013). A novel oral nutraceutical formula of omega-3 and omega-6 fatty acids with vitamins (PLP10) in relapsing remitting multiple sclerosis: a randomised, double-blind, placebo-controlled proof-of-concept clinical trial. *BMJ Open*.

[34] Jin H, Kanthasamy A, Ghosh A, Anantharam V, Kalyanaraman B, Kanthasamy AG (2014). Mitochondria-targeted antioxidants for treatment of Parkinson's disease: preclinical and clinical outcomes. *Biochimica et Biophysica Acta*.

[35] de Oliveira J, Hort MA, Moreira ELG (2011). Positive correlation between elevated plasma cholesterol levels and cognitive impairments in LDL receptor knockout mice: relevance of cortico-cerebral mitochondrial dysfunction and oxidative stress. *Neuroscience*.

[36] Palavra F, Marado D, Mascarenhas-Melo F (2013). New markers of early cardiovascular risk in multiple sclerosis patients: oxidized-LDL correlates with clinical staging. *Disease Markers*.

[37] Kardys A, Weinstock-Guttman B, Dillon M (2013). Cholesterol affects retinal nerve fiber layer thickness in patients with multiple sclerosis with optic neuritis. *European Journal of Neurology*.

[38] Weinstock-Guttman B, Zivadinov R, Horakova D (2013). Lipid profiles are associated with lesion formation over 24 months in interferon-*β* treated patients following the first demyelinating event. *Journal of Neurology, Neurosurgery and Psychiatry*.

[39] Murri M, Luque-Ramírez M, Insenser M, Ojeda-Ojeda M, Escobar-Morreale HF (2013). Circulating markers of oxidative stress and polycystic ovary syndrome (PCOS): a systematic review and meta-analysis. *Human Reproduction Update*.

[40] Kalyanaraman B (2013). Teaching the basics of redox biology to medical and graduate students: oxidants, antioxidants and disease mechanisms. *Redox Biology*.

[41] Refsum H, Smith AD, Ueland PM (2004). Facts and recommendations about total homocysteine determinations: an expert opinion. *Clinical Chemistry*.

[42] Bamonti F, Moscato GA, Novembrino C (2010). Determination of serum holotranscobalamin concentrations with the AxSYM active B12 assay: cut-off point evaluation in the clinical laboratory. *Clinical Chemistry and Laboratory Medicine*.

[43] Jacob KD, Hooten NN, Trzeciak AR, Evans MK (2013). Markers of oxidant stress that are clinically relevant in aging and age-related disease. *Mechanisms of Ageing and Development*.

[44] Aoyama K, Nakaki T (2013). Impaired glutathione synthesis in neurodegeneration. *International Journal of Molecular Sciences*.

[45] Rossi R, Dalle-Donne I, Milzani A, Giustarini D (2006). Oxidized forms of glutathione in peripheral blood as biomarkers of oxidative stress. *Clinical Chemistry*.

[46] Sharma SS, Dietz KJ (2009). The relationship between metal toxicity and cellular redox imbalance. *Trends in Plant Science*.

[47] Liu J, Qu W, Kadiiska MB (2009). Role of oxidative stress in cadmium toxicity and carcinogenesis. *Toxicology and Applied Pharmacology*.

[48] Ariu LNC, Morace G, Velio P, Campise M, Ciani A, Bamonti F (2006). Total Antioxidant Capacity (TAC) assay can be used as an environment toxicity index. *Clinical Chemistry*.

